# Application of the Complex Moments for Selection of an Optimal Sensor

**DOI:** 10.3390/s21248242

**Published:** 2021-12-09

**Authors:** Raoul R. Nigmatullin, Vadim S. Alexandrov

**Affiliations:** Radioelectronics and Informative-Measurement Technics Department, Kazan National Research Technical University Named after by A.N., Tupolev (KNRTU-KAI), K. Marx Str., 10, 420111 Kazan, Tatarstan, Russia; bridgelin2@yandex.ru

**Keywords:** complex moments, pressure sensors, multiple correlations

## Abstract

In the first time we apply the statistics of the complex moments for selection of an optimal pressure sensor (from the available set of sensors) based on their statistical/correlation characteristics. The complex moments contain additional source of information and, therefore, they can realize the comparison of random sequences registered for almost identical devices or gadgets. The proposed general algorithm allows to calculate 12 key correlation parameters in the significance space. These correlation parameters allow to realize the desired comparison. New algorithm is rather general and can be applied for a set of other data if they are presented in the form of rectangle matrices. Each matrix contains *N* data points and *M* columns that are connected with repetitious cycle of measurements. In addition, we want to underline that the value of correlations evaluated with the help of Pearson correlation coefficient (PCC) has a *relative* character. One can introduce also *external* correlations based on the statistics of the fractional/complex moments that form a complete picture of correlations. To the PCC value of internal correlations one can add at least 7 additional external correlators evaluated in the space of fractional and complex moments in order to realize the justified choice. We do suppose that the proposed algorithm (containing an additional source of information in the complex space) can find a wide application in treatment of different data, where it is necessary to select the “best sensors/chips” based on their measured data, presented usually in the form of random rectangle matrices.

## 1. Introduction and Formulation of the Problem

The widespread using of sensor systems in various technological and design solutions requires a more detailed assessment of the output parameters of physical quantities converted into an electric signal. It is necessary to understand exactly how the received signal is close to the real one, that is, it is always necessary to evaluate the final result containing a certain value of the hidden error. Now, a large number of solutions have been proposed that form the fundamental basis for selection of some efficient methods for processing the measured data [1,2,3,4,5,6,7,8,9,10]. In cases, when the analyzed data source is easily fitted to well-known analytical dependencies that follow from probability theory or from some proposed model, the calibration sensor problems do not arise usually. The situation becomes much more complicated with complex data arrays. In this case, it is not possible to establish the optimal distribution law due to the influence of many random factors. For solution of this problem in this complex case, the basic statistical methods of data processing, which are considered in brief below, are used.

**Statistical test method (Monte Carlo method)**. In this case, for estimation of some unknown parameters, it is necessary to carry out a set of tests and determine the arithmetic mean of the resulting values. It should be noted that as a result we get some approximate evaluation of the desired value with a certain amount of error due to the fact that repeating a series of experiments *N* times, according to the central limit theorem, will lead initial distribution to the normal one, however, the actual result can differ significantly from these random tests.

**The least squares method** is based on minimizing the sum of the squares of the deviations of the fitting functions (test data) from the original (reference data). This approach is quite simple for its implementation; however, it inevitably causes the following difficulties—the limited accuracy of the adjusted parameters and a strong binding to the time interval. This method also implies that the fitting function is known. However, in many cases it is not available.

**The maximum likelihood method** (based on the calculation of the joint sampling function) allows determining the parameters of the general population that gives the maximum likelihood function. The disadvantage of this approach is the fact that the distribution law of the data array must be known a priori. However, a researcher should keep in his mind that in the case of random data sequences they do not follow to well-known distribution laws. This basic requirement cannot be realized, especially in cases, when researcher deals with sequences that are close to trendless fluctuations/noise.

**Hypothesis testing (tests F, t, chi-square-distributions)**. In analysis of various types of data, a potential researcher makes some assumption about the correspondence (even remote and unjustified) of the studied dataset to a certain distribution law. Hypotheses are tested using various known criteria [1,2,3,4,5]. For example, the F-distribution is the ratio of two independent quantities, scaled relative to the original data. There is a good trend here—a specific transformation of the original dataset to the new key set. In the case, when it is necessary to set the scatter of some parameters (analysis of the variance), the Chi-square method [2,3] is used. In the case, when initial sampling is small, the Student’s t-test can be used to estimate the average of the selected values [1,2,3,4,5,6,7]. These methods work well in the theory of probability. However, in the case of the trendless sequences, it is necessary to evaluate data sets using other methods that are more accurate and reliable.

**Analysis of the evaluated variances and regressions**. In this approach it is necessary to take into account the influence of many external factors (input variables) on the parameters of the system studied (dependent variables), including the studied data that are considered as output variables. However, the calculation of these parameters is reduced to the central limit theorem, i.e., to normal distribution. In addition, as a priori statement about the absence of correlations and mutual influence of the parameters studied is supposed. Unfortunately, this supposition creates some uncontrollable error that is rather difficult to evaluate.

**Time series analysis**. This procedure is intended to eliminate the statistical fluctuation component and determines the initial dependence based on the controlled parameters [1,8,9,10]. However, in this case, a problem arises associated with the finding of the controlled (key) parameters that in many cases are remained as unknown. In addition, due to the finite time interval, it is not always possible to analyze the behavior of the function outside of the studied interval, which can introduce a significant amount of error. Therefore, it is advisable to move from time frames to discrete (counting) data points. The advantages and disadvantages of these basic methods are collected in Table 1.

Based on brief analysis of the conventional methods listed in Table 1, two general classes of *disadvantages* that are inherent in existing methods can be underlined:

(1) The presence of an uncontrolled model (in many cases it becomes difficult to evaluate the limits and drawbacks of the proposed model) and numerous treatment errors due to the selection of the mathematical method and an approximate evaluation of a random variable.
(2) The requirement a priori knowledge of the distribution law for a set of the chosen random variables and the reduction in the investigated data array to the normal distribution law, guided by the central limit theorem. In real situations it is difficult to realize the requirements of this theorem and a potential researcher does not have conditions for its verification.

This brief analysis of the existing methods allows to put forward the following question:

*Is it possible to suppose a “universal” method that is free from the model assumptions and treatment errors and can be applied to any set of random functions?*

This method should contain an additional source of information in another space. We mean that besides temporal and frequency conventional spaces associated by the Fourier transformation one can introduce a space of complex numbers associated with complex moments. This method also should evaluate a set of desired correlations that follow from independent expressions. From our points of view, the answer lies in generalization of the well-known Pearson correlation coefficient (PCC) and the existing concept of the integer moments. In this paper, we make the further step and propose the concept of the *complex* moments that give us four additional mathematical expressions that will be suitable for independent evaluations of the external correlations. These expressions can be considered as an additional source of information for evaluation of the desired correlations. In addition, with the help of independent evaluations of the ranges (see expression (12) below) one can compare all independent correlations together and choose the optimal sensor based on the statistical analysis of the measured pressure data.

The content of the paper is organized as follows. In the second chapter we propose the theory of the complex moments and obtain useful expressions for the further data treatment. In the third chapter we give the measurement details related to receiving of the desired set of data. In the fourth chapter we propose the treatment algorithm and obtain the necessary results. In the final chapter, we make necessary conclusions and discuss some details that can be useful for potential researches that will deal with their own random data.

## 2. Statistics of the Complex Moments

The correlation analysis plays a key role in data signal processing. Any manual related to the conventional statistics, many other books and papers are related to consideration of this important subject [11,12,13,14,15,16,17,18,19]. Why this type of analysis is significant? The evaluation of different correlations is important especially in complex systems, where the fitting function that can be derived from a simple model in many cases is *absent*.

The first generalization from the integer moments to a set of the fractional moments has been done in paper [20] and then was applied successfully for available data [21,22,23].

(1) $$\cos\left(\theta \right)=\frac{\left(y_{1}y_{2}\right)}{\sqrt{{\left(y_{1}\right)}^{2}{\left(y_{2}\right)}^{2}}},\;\left(y_{1}y_{2}\right)=\sum_{j=1}^{N}\left(y_{1}\left(j\right)y_{2}\left(j\right)\right)$$

However, attentive analysis shows that the conventional Pearson correlation coefficient (PCC) cannot cover all variety of “resemblances” that exist between the compared random sequences. From our point of view, the value of pair correlations obtained with the help of expression (1) has a *relative* character. Generalization of this important formula, describing some internal correlations becomes possible if one generalizes the concept of the integer moments and introduce the *fractional* moments that covers all admissible interval of the real moments. In papers [21,22,23] and in the recent book [24] one of the authors (RRN) introduced the definition of the generalized Pearson correlation function (GPCF) for a pair of the chosen random sequences *y*_1_ (*j*), *y*_2_ (*j*) (*j* = 1, 2, …, *N*) based on the generalized mean value function *G_p_* (*y*_1_, *y*_2_)

(2) $$\begin{array}{l} GPCF_{p}=\frac{G_{p}(y_{1},y_{2})}{\sqrt{G_{p}(y_{1},y_{1})\cdot G_{p}(y_{2},y_{2})}},\;\\ G_{p}(y_{1},y_{2},\ldots ,y_{k})={\left(\frac{1}{N}{\sum_{j=1}^{N}\left|yn_{1}\left(j\right)\cdot yn_{2}\left(j\right)\cdot \ldots \cdot yn_{k}\left(j\right)\right|}^{mom_{p}}\right)}^{\frac{1}{mom_{p}}} \end{array}$$

Here, a set of the functions *yn_k_* (*j*), *k* = 1, 2, …, *K* defines the normalized values of the compared sequences located in the interval [0, 1]. The normalized value of the function *yn* (*j*) is defined as

(3) $$yn(j)=\{\begin{array}{l} \frac{y_{j}+\left|y_{j}\right|}{\max(y_{j}+\left|y_{j}\right|)}-\frac{y_{j}-\left|y_{j}\right|}{\min(y_{j}-\left|y_{j}\right|)}\\ \frac{y_{j}-\min(y_{j})}{\max(y_{j}-\min(y_{j}))}\;(\mathrm{for}\;\mathrm{initial}\;\mathrm{positive}\;\mathrm{sequence}) \end{array}$$

The values of the moments are taken from interval

(4) $$mom_{p}=\exp\left(-r+2\frac{p}{P}r\right),\;e^{-r}\leq mom_{p}\leq e^{r},\;p=0,1,\ldots P$$

The chosen interval in (4) for the set of the moments covers practically all positive values of the real moments and, therefore, can be considered as an optimal one. Usually, the limiting values of *r* in (4) are taken from the interval (10–15) and this selected interval is sufficient for evaluation of all possible correlations that are given by the expression (2). We should notice also that (2) being located in the interval [0, 1] has “universal” behavior. At negative values of the argument *mom_p_* it tends to the unit value, at positive values of the *mom_p_* it achieves the limiting value *L* at *p* = *P*. The value *L,* in turn, can accept three values: (a) *L* = 1 that corresponds to the case of strong correlations; (b) the interval *M* < *L* < 1 corresponds to the case of intermediate correlations; (c) the case *L* = *M* (*M* is minimal value of (2)) corresponds to the weak correlations. We should notice also that weak correlations *always* exist and the case of the absence of correlations between two random sequences can be considered as a *supposition*. However, the case of the absence of correlations can be prepared *artificially* if one can use the orthonormalized Gramm-Schmidt procedure, widely used in quantum mechanics. This “universal” behavior of the function (2) allows to determine the complete correlation factor as the following product

(5) $$CF=L\cdot M,\;M^{2}\leq CF\leq M.$$

The complete correlation factor CF can characterize the degree of correlation between a couple of the chosen random sequences. The analysis based on expressions (2) and (4) was tested on many real and mimic data [11,22,23,24]. In addition to expression (4), one can introduce the parameter that defines a class of correlations described above.

(6) $$Cls=\frac{L-M}{1-M}$$

If *L* = 1 and *M* ≠ 1 and 0.8 ≤ Cls ≤ 1 then *CF* can be referred to high correlations (HC) case. When *L* ≅ *M* and 0 ≤ Cls < 0.2 then CF is referred to the low correlations (LC) case. When Cls occupies some intermediate position (0.2 < Clf < 0.8) then we deal with intermediate correlations (IM) case. We should note also the case *M* = 1, when the minimum of (2) is absent and the compared functions are identical to each other. In this case, we put Cls = 0/0 = 0.

However, in order to close the problem with correlations it is necessary to make the next step and consider the generalized Pearson correlation function based on the total set of the moments, including their complex parts. Therefore, we define the generalized Pearson correlation function for the *complex* moments (GPFCM) by the following expression

(7) $$\begin{array}{l} GPFCM_{p}=\frac{GMVC_{p}(y_{1},y_{2})}{\sqrt{GMVC_{p}(y_{1},y_{1})\cdot GMVC_{p}(y_{2},y_{2})}},\;\\ GMVC_{Z_{p}}(y_{1},y_{2},\ldots ,y_{k})={\left(\frac{1}{N}{\sum_{j=1}^{N}\left|yn_{1}\left(j\right)yn_{2}\left(j\right)\ldots yn_{k}\left(j\right)\right|}^{Z_{p}}\right)}^{\frac{1}{Z_{p}}} \end{array}$$

In expression (7), the initial set of random sequences *yn*_1_ (*j*), *yn*_2_ (*j*),…, *yn_k_* (*j*) are normalized to the interval [0, 1] in accordance with expressions (3), the value of *Z_p_* accepts *complex* values; it contains two parts and located in the following intervals:

(8) $$\begin{array}{l} Z_{p}=R_{p}+i\Omega_{p},\;R_{p}=\exp\left(-L+2L\frac{p}{P}\right),\;\Omega_{p}=\Omega_{0}+\frac{p}{P}\left(\Omega_{L}-\Omega_{0}\right),\\ e^{-L}\leq R_{p}\leq e^{L},\;\Omega_{0}\leq \Omega_{p}\leq \Omega_{L},\;p=0,1,\ldots ,P. \end{array}$$

In order to separate the real and imaginary parts from numerator of expression (7) we introduce the following sums

(9) $$\begin{array}{l} Sr_{p}(1,2)=\frac{1}{N}\sum_{j=1}^{N}{\left(yn_{1}\left(j\right)yn_{2}\left(j\right)\right)}^{R_{p}}\cos\left[\Omega_{p}\ln\left(yn_{1}\left(j\right)yn_{2}\left(j\right)\right)\right],\\ Sm_{p}(1,2)=\frac{1}{N}\sum_{j=1}^{N}{\left(yn_{1}\left(j\right)yn_{2}\left(j\right)\right)}^{R_{p}}\sin\left[\Omega_{p}\ln\left(yn_{1}\left(j\right)yn_{2}\left(j\right)\right)\right]. \end{array}$$

With the help of expressions (9) the numerator of expression (7) can be presented in the form.

(10) $$\begin{array}{l} G_{Z_{p}}(1,2)={\left(\frac{1}{N}\sum_{j=1}^{N}{\left(yn_{1}(j)yn_{2}(j)\right)}^{Z_{p}}\right)}^{\frac{1}{Z_{p}}}=\left|G_{Z_{p}}(1,2)\right|\exp(i\Phi_{p}(1,2))\\ \left|G_{Z_{p}}(1,2)\right|=\frac{\exp\left[\ln\left|S_{p}\left(1,2\right)\right|\right]}{\left|Z_{p}\right|},\;\Phi_{p}(1,2)=\varphi_{p}\left(1,2\right)-\tan^{-1}\left(\frac{\Omega_{p}}{R_{p}}\right). \end{array}$$

The values $\left|S_{p}(1,2)\right|,\varphi_{p}(1,2)$ are determined with the help of expressions (9) as

(11) $$\left|S_{p}\left(1,2\right)\right|=\sqrt{Sr_{p}^{2}\left(1,2\right)+Sm_{p}^{2}\left(1,2\right)},\;\varphi_{p}(1,2)=\tan^{-1}\left(\frac{Sm_{p}\left(1,2\right)}{Sr_{p}\left(1,2\right)}\right)$$

Expressions in dominator figuring in (5) are obtained easily with the help of last expressions (7)–(9), where it is necessary to replace the corresponding sequences (1↔2). Finally, expression (5) after separation of the real and imaginary parts can be presented in the form

(12) $$GPFCM_{p}=\frac{\left|G_{Z_{p}}(1,2)\right|}{\sqrt{\left|G_{Z_{p}}(1,1)\right|\cdot \left|G_{Z_{p}}(2,2)\right|}}\exp\left(i\left[\Phi_{p}(1,2)-\frac{1}{2}\left(\Phi_{p}(1,1)+\Phi_{p}(2,2)\right)\right]\right)$$

Other expressions, entering into (12) are defined by expressions from (10). Expression (12) represents itself the final formula that generalizes the previous results obtained earlier in papers [21,22,23,24] for the case of real fractional moments. With the help of new expressions (10)–(12) we want to show that possible generalization of the concept of correlations based on the conventional definition (1) has a *relative* character. It means that correlations from the fixed number is transformed to a distribution; they can be arbitrary and occupy all admissible interval [−1, 1] and even exceed it. These expressions will be used in Section 4 for evaluation of different types of correlations that are given by complex moments.

Finishing this section, we want to stress one important point. The concept of the complex moments alongside with fractional moments introduced earlier enriches the reduced “feature” space by additional set of independent parameters and can help in differentiation of the “hidden” differences, when the conventional methods do not “see” them. This solution becomes possible because the concept of the complex moments contains additional formulae (10) and (12). They can be helpful in selection of the “hidden” differences in these complex cases.

## 3. Experimental Scheme and Measurements Details

**Figure 1:**
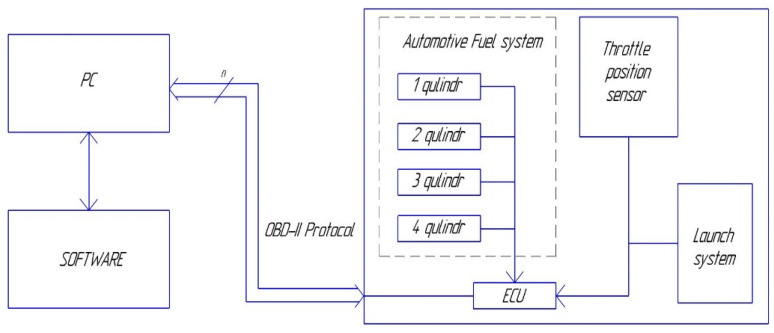
Block diagram explaining the measured procedure of experimental data.

The collection of experimental data was carried out in accordance to the scheme shown in Figure 1.

Ten sensors were considered as initial data. Their principle of operation is based on the conversion of the control mechanical pressure into an electrical signal. These measuring devices are installed in the fuel rail of the automotive system and are designed to regulate the fuel supply to each cylinder. Note that sensors 1 and 3 operate in symmetrical mode (odd channels) similar to sensors 2 and 4 (even channels). Therefore, their output signals can be expected to be same. For registration the desired data, a personal computer with preinstalled Car Scanner software and appropriate drivers were used. The connection was made using the OBD-II protocol, which is currently installed in all similar vehicles. To obtain more reliable information, the connection was made using a tested cable, excluding a wireless connection.

The cable was connected and the program was started with the car ignition off (the output signal from the sensors is absent). When the ignition is turned on, the output signal registered by the sensors is proportional to the engine speed. Further measurements were made only after the so-called “warm-up revolutions”, i.e., when “the establishing idle speed” state was achieved. By adjusting the throttle position with using the accelerator pedal, the amount of air supplied to the combustion chamber was changed. As it is known, the fuel mixture has a certain ratio and this ratio is kept as unchanged. Consequently, the changing in the angle of the flap position entails a change in the amount of fuel supplied to the given injector, which affects the output readings of the sensors in each cylinder. To obtain a more accurate result, the same experiment was repeated 10 times. As an output signal, one can obtain an array of data, which was written to a computer file through an electronic control unit (ECU). As a next step it is necessary to analyze the data and compare them with each other. We paid a special attention to operation of sensors working in symmetric modes.

Our particular interest is the operation of sensors 2 and 4, operating in a symmetric mode. The process of diagnosing fault is coded by the system of the ECU. It is turned out that a lean mixture is controlled by the channel No. 2, therefore, an error of the corresponding type has been preserved in the memory of the computer system, which was displayed both on the indicator block as a “check engine”.

When comparing several elements with each other, such concepts as “identical”, “similar”, “different”, “completely different”, etc., arise, which is usually estimated by some quantitative value, called a statistical error, or some fitting error. In the event that this error value is low/high enough, it is necessary to make a decision about which of the tested elements is considered a priori as the reference one (i.e., it works in the mode established by the manufacturer with a high value of the considered parameters), and which sensor will be tested one (subject of verification).

In our case, sensor number 4 in channels 2–4 can be considered a priori as the reference, since it does not generate errors, and the sensors 2 including all measured set (0–9) can be considered as the tested ones, since there is an error in the system memory connected with the selected cylinder. However, important question related to selection of the “reference” sensor is remained. What kind of the justified arguments one can add in order to select the desired reference sensor?

In the symmetrical system 2–4, the choice of selection of sensor 4 can be argued as follows: the message about the malfunction of the second cylinder was recorded in the memory of the electronic control unit (ECU) as the error P0171 “Too poor fuel-air mixture”, which persisted for a long period of time. Therefore, for the analysis of the real state of channel 2, this sensor was accepted as a test one. The discrepancy of the parameters was confirmed based on a superficial analysis of the studied data using the built-in Pearson correlation coefficient (PCC). In addition, with the help of a more sensitive method associated with the calculation of fractional and complex moments, there is a chance to establish more noticeable and significant differences in the studied channels operating in symmetric modes. The treatment algorithm is given below in Chapter 4.

To evaluate and confirm the high efficiency of the described method, we apply a similar analysis for channels 1–3 and establish, as it was assumed, a high degree of correlation compared to the previously described symmetric channel. Our assumptions were confirmed. Since there was no information about the malfunction of any sensor (1 or 3) in the system, the choice of the reference variant is remained questionable because in the symmetric mode state the sensors 1–3 do not demonstrate significant differences. However, it should be noted that in order to obtain a significant result, it is necessary to choose a reference and test options based on some quantitative parameters.

In our opinion, such parameters can be short-term fuel correction time (STFCT) (refers to instantaneous changes in the fuel mixture) and long-term fuel correction time (LTFCT) (shows the change in the fuel mixture over a long period of time based on the indications of short-term correction). These parameters can be obtained by connecting a diagnostic scanner according to the scheme (see Figure 1). It should be noted that the change of these parameters in the ECU memory occurs infrequently, and during a series of 10 tests they remained constant, therefore, they can be considered as a stable. Is it possible to explain what kind of quantitative parameters can characterize this stable state? At the moment of time, when the fuel–air mixture burns oxygen sensor located in the exhaust system of the car determines the content of the given mixture presented in greater quantities: fuel or air. It should be noted that the assessment of the readings by the oxygen sensor would be completely different from the sensors considered earlier, since the correction system is located in a completely different node of the car. If all the fuel and air are burned in the required ratio, then the correction factor gives zero percentage. If an excess of fuel is detected, then a correction with a “plus” sign occurs, if the excess of air is a correction with a “minus” sign for two parameters LTFCT and STFCT, accordingly. By evaluation of the arithmetic mean of these parameters, it becomes possible to identify which of the channels is less affected by the found correction. Therefore, if it operates in a more stable mode then it is considered as a reference. For the sensors under consideration, the values of the estimated parameters are listed in Table 2. Since it is not known exactly which values should be used, the current results (taking into account the sign) and absolute values should be analyzed.

Analyzing the results given in Table 2 for two given channels, it can be seen that the correction value for the sensor number 1 is smaller (taking into account the sign) and its absolute value. Based on this observation, we made a conclusion that the sensor number 1 will be the reference, and the sensor number 3 will be the tested sensor. It should be noted that the calculation results are turned out to be quite close, which allows us to conclude that the given sensors operate in symmetrical modes. Nevertheless, the problem of selection some reference sensor is remained and this problem should be solved in each private case independently. Here, we demonstrated a possible solution.

## 4. Proposed Algorithm and Data Treatment Procedure

The key problem that is followed from the statistics of the complex moments can be formulated as:

*What kind of new informative component is added by imaginary part of the complex moments in a feature space for evaluation of correlations (external and internal ones) more accurately (free from model and treatment errors) in comparison with the conventional PCC and other methods?*

These new “informative units’ extracted from complex moments should be helpful in differentiation of initial data and detection of possible correlations between columns, if initial data are presented usually in the form of rectangle *N* × *M* matrix having *N* rows (*j* = 1, 2, …, *N*—number of data points) and *m* = 1, 2, …, *M* (number of columns). More specifically, we want to demonstrate the solution of the following problem:

Is it possible to compare a couple of random functions in the space of fractional and complex moments and receive more adequate functions, pretending on a more accurate “resemblance” with each other? This problem is facilitated if one of the random functions is chosen as the *reference* one. For solution of this problem, it is necessary to normalize the initial data and make them completely dimensionless and close to each other with the help of expression

(13) $$\begin{array}{l} yn_{j}=\frac{y_{j}-\langle y\rangle }{Range(y)},\;\langle y\rangle =\frac{1}{N}\sum_{j=1}^{N}y_{j},\\ Range(y)=\max(y)-\min(y),\\ Range(yn)=1. \end{array}$$

For comparison of two random functions, we use a simple expression that can be used for additional evaluation of *external* correlations

(14) $$Ecr(y_{i},y_{j})=\frac{Range(y_{i})+Range(y_{j})}{\max(y_{i},y_{j})-\min(y_{i},y_{j})}$$

Any random function *y* is located in the rectangle with the sides: [*Range* (*x*) = max (x) − min (x) that coincides with horizontal direction and *Range* (*y*) = max (*y*) − min (*y*) that coincides with the height of rectangle in the opposite direction]. If the function of external correlations *Ecr* (*y_i_*, *y_j_*) lie in the interval [1, 2] then the compared functions (read rectangles) cross each other. If this correlation function *Ecr* (*y_i_*, *y_j_*) lies in the interval (0, 1) it means that a couple of two compared functions (*y_i_*, *y_j_*) do not cross each other, and, therefore, are *uncorrelated*. Expression (12) is turned to be useful for evaluation of *external* correlations of different random functions, especially in cases when the fitting function following from some proposed model is *absent*.

**Figure 2:**
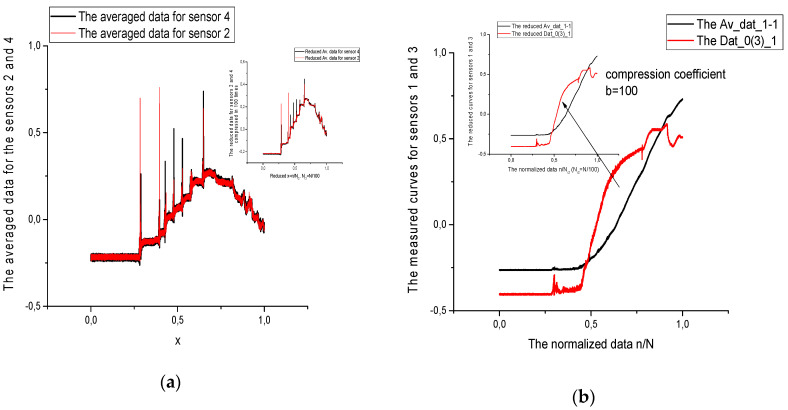
(**a**) On the left. Here, we plot the initial data containing 263,600 data points for the averaged data associated with reference sensor (bolded line). Red line corresponds to the averaged data for sensor −2. In the right corner above we place the reduced data for the same data compressed in b = 100. The correlation coefficient equals 0.987. They exhibit the scaling/fractal properties, from one side, and accelerate the calculations, from another side. Vertical axis gives the value of response in Volts. Horizontal axis is dimensionless and the measured data points are normalized to the unit value. (**b**) Data for the reference function (bolded black line) are shown on the right. The red line depicts the data for sensor 0 (3). In the left corner of this figure we depict the same data compressed in *b* = 100 times. As it was marked in Figure 2a, vertical axis gives the value of response in Volts and horizontal axis is dimensionless and the measured data points are normalized to the unit value.

**Figure 3:**
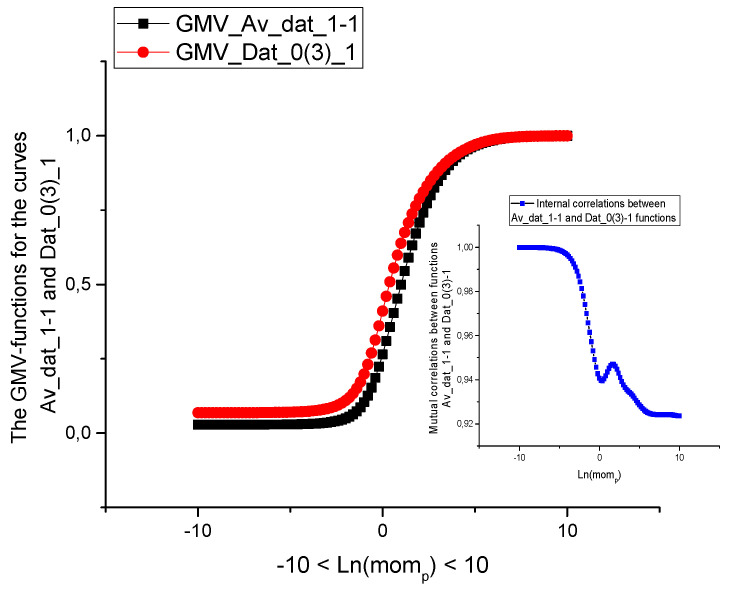
In the central figure we compare the GMV-functions for the same curves Av_dat_1-1 and Dat_0 (3) calculated in the space of the fractional moments. In the right corner below we depict the generalized Pearson correlation function (Equation (2)) in the space of the fractional moments placed in the same interval [−10, 10].

We choose the following correlation parameters having in mind that true value of correlation has a *relative* character. These parameters are defined below and used as notions in Table 3, Table 4 and Table 5 (for sensors 2–4) and Table 6, Table 7 and Table 8 (for sensors 1–3), where the chosen data, corresponding to the given pressure sensors are compared with each other. We should note also that initial data can be compressed by means of procedure of reduction to three incident points, that was widely used earlier in papers [25,26]. This procedure is simple and it is used for testing the similarity of initial data under the compression. In addition, it decreases the computational cost and keeps all basic peculiarities that are necessary for the further data processing. In our case the compression parameter *b* = 100. The Figure 2 and Figure 3 demonstrate the effectiveness of this procedure. It shows that in many cases the measured curves are self-similar/fractal. The reduced curves (compressed in 100 times) are placed in small figures. This procedure reduces also the computational cost and keep the basic peculiarities of the data considered.

We chose the averaged data from all sensors of the type 4 and accepted these data as the reference ones. The different sensors referred to the class 2 are considered as the tested ones. We have again the averaged data taken from all over available data belonging to class 2. In addition, we have 10 separate datasets obtained for different sensors, belonging to class 2. The same procedure was realized for another class of sensors 1–3. For this class, the average value for the class—1 is chosen as the reference one, other data referred to class 3 are considered as the tested sensors.

In order to analyze deeply all possible correlations and possible deviations from the reference data we propose the following set of parameters:

CP_1_(*s*)—direct calculation of external correlations with the help of expression (12) for the normalized data *yn* (*s*) obtained from (11);

CP_2_(*s*)—calculation of external correlations for a couple of GMV-functions. These functions are evaluated with the help of (2a);

CP_3_(*s*)—calculation of the complete correlation factor (CF) based on internal correlations. The factor is evaluated from expression (4a);

CP_4_(*s*)—Pearson correlation parameter (PCC). It is defined by expression (1);

CP_5_(*s*)—calculation of correlation parameter that determines the class of correlations. For its evaluation we use expression (4b);

CP_6_(*s*)—calculation of the ranges for the differences *DF*_s_ = *y*_s_ − *y*_1_, where *y*_1_ = Av_dat_4 (chosen as the refence data) and *y*_s_= Dat_s (2), *s* = 0, 1, …, 9—a set of the tested data. For sensors 1–3 *y*_1_ = Av_dat_1 and *y_s_* = Dat_s (3);

CP_7_(*s*)—external correlation parameter that corresponds the comparison of the absolute values of the complex moments for the pattern functions *y*_1_ = Av_dat_4 (*y*_1_ = Av_dat_1) with others *y*_s_= Dat_s (2), (*y_s_* = Dat_s (3)) for *s* = 0, 1, …, 9. It is evaluated for two module functions $\left|G_{Z_{p}}\left(y_{1},y_{1}\right)\right|$ and $\left|G_{Z_{p}}\left(y_{s},y_{s}\right)\right|$ from (8). We use the same notations as they are used above;

CP_8_(*s*)—external correlations for comparison of two phases $\Phi_{Z_{p}}(y_{1},y_{1})$ and $\Phi_{Z_{p}}(y_{s},y_{s})$, obtained in the frame of the complex moments;

CrP_9_(*s*)—external correlations of the absolute values $\left|G_{Z_{p}}\left(y_{1},y_{s}\right)\right|$ and $\sqrt{\left|G_{Z_{p}}\left(y_{1},y_{1}\right)\right|\cdot \left|G_{Z_{p}}\left(y_{s},y_{s}\right)\right|}$ obtained in comparison of pair correlations for the complex moments;

CrP_10_(*s*)—external correlations of the phase parts $\Phi_{Z_{p}}(y_{1},y_{s})$ and $\frac{1}{2}\left(\Phi_{Z_{p}}(y_{1},y_{1})+\Phi_{Z_{p}}(y_{s},y_{s})\right)$, obtained for the complex moments;

CrP_11_(*s*)—ranges of the absolute values for significant difference *y_s_* − *y*_1_;

CrP_12_(*s*)—ranges of the phase parts for significant difference *y_s_* − *y*_1_.

These 12 quantitative parameters (actually the functions *y_s_*) are turned to be useful for receiving a set of the desired correlations. These correlations are sufficient in order to select the *maximal* values of parameters CrP_1–5_, CrP_7–10_ from the interval [1,2] and *minimal* deviations for parameters CrP_6,11,12_. If any *y_s_* will contain a maximal number from all compared set *s* = 0, 1, …, 9) then it can be admitted as the “best” one from the statistical point of view. If any parameter CrP_1–12_ pretending to be optimal has minimal number of the desired “scores” then one includes the values of the standard deviations for extending the desired selection interval [max (*y*), max (*y*) − stdev (*y*)] (for the parameters CrP_1–5_, CrP_7–10_) and [min (*y*), min (*y*) + stdev (*y*)] (for parameters CrP_6,11,12_). This procedure helps to find the closest correlations that can form the “best” class. The “best” selected sensors (their correlated values bolded) are presented in Table 5 and Table 8.

The meaning of the chosen correlation parameters presented in Table 3, Table 4, Table 6 and Table 7 are explained above.

Finishing this section, it is necessary to show a universal criterion that was used in selection the closest correlations. In each column we choose three/four maximal correlations that are close to each other. Then, these three correlations are compared with the values from different columns located in each line. The maximal number of correlators and minimal number of ranges (for parameters CrP_6,11,12_) located in each line helps to choose the optimal sensor.

In these tables, we underlined the most probable values that are closed to maximal values for parameters CP_1–4_ and CP_7–10_. As for CP_6_,_11–12_ we underlined the parameters that are close to minimal deviations. Obviously, we excluded the first two rows in these tables because they demonstrate an “ideal” case (the first row) and effectiveness of the averaged procedure (the second row). All bolded values for parameter CP lie in the interval max(CP) − stdev(CP) and min(CP) + stdev(CP). We choose the following criterion for selection of the best pressure sensor: maximal bolded numbers in each row. In accordance with this criterion, we obtain the following optimal selection that is given in Table 5 (sensors 2) and Table 8 (sensors 3).

The meaning of the rest correlation parameters presented in Table 3 are explained in the text also. Here and below the bolded numbers coincide with maximal values in columns **CP_1_**–**CP_5_** and minimal value for **CP_6_**. The maximal/minimal value in addition is underlined. 

Based on parameters that are given in Table 5 one can conclude that the first place (“golden medal”) belongs to sensors 9 and 6; the second place (“silver medal”) can be divided between sensors 7, 8 and 3 and finally, the third place (“bronze medal”) belongs to sensors 1 and 2.

The meaning of the rest correlation parameters presented in Table 7 are explained in the text also.

Based on parameters that are given in Table 8 one can conclude that the first place (“golden medal”) belongs to sensor 9; the second place (“silver medal”) can be divided between sensors 3 and 6 and finally, the third place (“bronze medal”) belongs to sensor 5.

**Figure 4:**
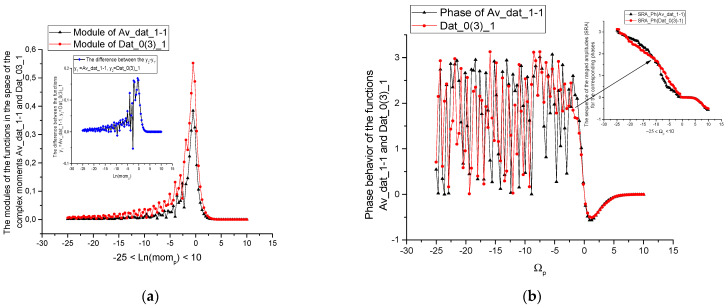
(**a**). The behavior of the initial functions (see Figure 2 above) in the space of the complex moments. Here, the corresponding modules are shown. On the left corner above we show the difference between these functions. (**b**). The behavior of the initial functions (see Figure 2 above) in the space of the complex moments. Here, the corresponding phases are shown. On the left corner above we show the sequences of the range amplitudes calculated for these functions. These functions are sensitive for evaluation of the differences between them.

In addition, we demonstrate the Figure 2, Figure 3 and Figure 4, which explain the behavior of the reduced curves (shown in small Figure 2) in the space of the fractional (Figure 3) and complex moments (Figure 4a,b), accordingly. As one can see from these figures the behavior of these curves is rather “universal” Figure 4a corresponding to the module of the complex moments forms a “resonance” curve with maximum near “zero” moment Ln(*mom_p_*). The phase behavior depicted in Figure 4b is also “universal”. This curve demonstrates strong oscillations for negative values of the Ω*_p_* and tends to zero for positive values of Ω*_p_*. This behavior allows to select fractional/complex moments for analysis of a wide class of random curves.

## 5. Discussion and Basic Conclusions

In this paper, we apply the statistics of the complex moments for evaluation of the additional correlations. Finishing the final section one can make the following conclusions:

1. We propose some “universal” tool as a source of additional information for detection of “hidden” correlations in many complex systems when the specific model is absent and, therefore, it is difficult to differentiate the desired differences;
2. For reliable evaluations the values of internal correlations the conventional PCC is not sufficient. As one sees from the paper the statistics of the fractional/complex moments allows to divide the correlations on two independent classes: (a) external correlations that allow to compare the samplings having different or equal number of data points; (b) internal correlations that allow to compare the samplings having equal number of data points only. As it follows from this analysis the concept of correlation has a *relative* character. It becomes impossible to receive their absolute values;
3. The method proposed in this paper is *free* from the model and treatment errors and has rather *general* character. It can be applied to *any* set of data (having a trend or without one);
4. The algorithm of application of this statistic is described in Section 4. We should stress here usefulness of expressions (13) and (14) that make initial data dimensionless and close to each other. Expression (14) for Ecr(*y*_1_,*y*_2_) has also universal character. It can be applied for comparison of any couple of random functions located in the given interval. We should stress here that combination (Ecr(*y*_1_,*y*_2_) − 1) 100% gives the degree of overlapping between two functions *y*_1_ and *y*_2_; 100%—corresponds to the complete “fusion”, while the value ≅ 0% signifies the absence of fusion. If this combination becomes negative then it shows the degree of “disconnection” between two compared functions;
5. The criterion for selection of the “best” sensor is described in the previous Section 4. This criterion is rather universal and can be applied to *any* data presented in the form of rectangle matrix. Each column of this matrix describes the independent correlations given by the statistics of the fractional/complex moments, while each line of this matrix belongs the compared part. In our case a “part” is associated with the sensor;
6. As it follows from analysis of specific data presented in Table 3, Table 4, Table 6 and Table 7 one can apply 6 external and independent correlations (CP_1–2_, CP_7,8,9,10_) and couple of internal correlations (CP_3–4_) for reliable selection of an “optimal” sensor based on its correlation characteristics. In addition, one can evaluate the ranges of the relative differences (CP_6,11,12_) between the tested and reference data that are turned to be useful for selection of the “best” sensor, as well.

Finishing this final section one can say that a potential researcher receives a new and general tool for analysis of different data, especially in cases when it is necessary to compare a couple of random functions and select the “best” one if one of the compared functions is considered as the reference/pattern function.

## Figures and Tables

**Table 1:** The conventional statistical methods that are used in information processing.

NO.	The Name of the Method	Advantages	Disadvantages
1	The Monte-Carlo method	Simple structure of the computational algorithm. The possibility of application for a wide class of mathematical models.	The occurrence of an uncontrolled error due to an approximate evaluation of a random variable; Obtaining an acceptable result is possible only as a result of a multiple increase in the number of tests.
2	The least square method	Easy in implementation; It has a “universal” computational procedure.	Limited accuracy for the customized parameters; Strong binding to the selected time interval.
3	Maximum-likehood method	Allows to register and process both grouped and non-grouped information; The low value of the variance parameter and, accordingly, the standard deviation	The needing a priori information about the distribution law of the data array, which, for example, is difficult to apply to the selected trendless sequences; A significant number of calculations is required.
4	Hypothesis testing (chi-square, F-test, *t*-test)	Allows to verify and describe the proposed hypothesis with a high degree of accuracy for most data types. Usually, these hypotheses are close to the Gaussian distribution.	The occurrence of an uncontrolled error due to the insensitivity of the method to data containing trendless noise.
5	Analysis of variances	Allows to test more complex hypotheses compared to the method described above due to factor analysis.	A priori statement about the absence of correlation and mutual influence of the studied parameters on each other.
6	Regression analysis	Allows to get an acceptable result of the adjusted data relative to the initial data for a finite time interval if a limited set of factors under study is taken into account.	The occurrence of an uncontrolled error due to the inability to take into account the basic external factors on which the output value depends.
7	Time-series analysis	Allows to determine the initial dependence on the controlled variable with a high degree of accuracy.	The problem associated with the finding of controlled (key) parameters; For the fixed and finite time interval, it is not possible to analyze the behavior of the function beyond the boundary of the studied interval. This attempt is accompanied by significant amount of the uncontrolled error.

**Table 2:** Values of STFT and LTFT parameters registered for sensors 1 and 3.

No of Sensor	STFT	LTFT	(STFT + LTFT)/2	|STFT + LTFT/2|
1	+0.04%	−0.02%	0.01%	0.03%
3	+0.06%	+0.04%	0.05%	0.05%

**Table 3:** The comparison of different pressure sensors for selection the optimal one between sensors 2. The sensor 4 is chosen as the pattern one.

Parameters	CP_1_	CP_2_	CP_3_	CP_4_	CP_5_	CP_6_
Av_dat_4-4	2.00000	2.00000	1.00000	1.00000	0.00000	0.00000
Av_dat_2-4	1.92061	1.98463	0.99090	0.99476	1.00000	0.55556
Dat_0(2)-4	1.70812	1.87657	0.35850	0.50096	0.00000	0.73878
Dat_1(2)-4	**1.77456**	**1.97723**	**0.51981**	0.77936	0.00000	0.82199
Dat_2(2)-4	**1.82209**	**1.98434**	**0.42453**	0.76471	0.00000	0.59856
Dat_3(2)-4	1.64490	1.90466	0.42725	**0.91952**	0.00000	**0.55907**
Dat_4(2)-4	1.70848	1.89775	0.01748	0.41120	0.00000	1.00556
Dat_5(2)-4	1.70610	1.89553	0.08288	0.75774	0.03199	0.90528
Dat_6(2)-4	**1.84175**	**1.95250**	0.35258	**0.91273**	0.00000	0.77201
Dat_7(2)-4	1.64295	1.94093	**0.75510**	0.60961	1.00000	**0.52327**
Dat_8(2)-4	1.58442	1.93771	0.38956	**0.89027**	0.00000	**0.50330**
Dat_9(2)-4	1.64399	**1.95634**	**0.46726**	**0.93791**	0.00000	**0.45556**

**Table 4:** Additional correlation parameters that were extracted from complex moments.

Parameters	CP_7_	CP_8_	CP_9_	CP_10_	CP_11_	CP_12_
Av_dat_4-4	2.00000	2.00000	2.00000	2.00000	0.00000	0.00000
Av_dat_2-4	1.97672	1.96094	1.62537	1.96052	0.14781	0.62314
Dat_0(2)-4	1.89042	1.97025	1.43929	1.78006	**0.32158**	**0.37876**
Dat_1(2)-4	1.80958	1.91885	1.55008	**1.93186**	0.50223	0.51391
Dat_2(2)-4	1.85315	**1.94167**	1.47640	1.84744	0.39057	**0.45681**
Dat_3(2)-4	**1.94629**	**1.95683**	**1.61756**	1.87538	**0.35610**	0.50784
Dat_4(2)-4	1.55217	1.91368	1.15797	1.80164	0.42078	0.95679
Dat_5(2)-4	1.55215	1.89460	1.17895	**1.91341**	0.42383	0.99566
Dat_6(2)-4	**1.96605**	1.91791	**1.58189**	**1.94122**	**0.37310**	0.52606
Dat_7(2)-4	1.64515	1.83867	**1.59583**	**1.92842**	**0.39639**	1.11090
Dat_8(2)-4	**1.99556**	**1.97457**	1.48013	1.83007	0.42879	**0.36855**
Dat_9(2)-4	**1.96199**	**1.92880**	**1.55389**	1.91143	0.41355	0.53128

**Table 5:** The selection of the best sensor among sensors numbered 2.

The Available Set of the Selected Pressure Sensors	Number of Optimal Parameters
Dat_0(2)-4	2
Dat_1(2)-4	**4**
Dat_2(2)-4	**4**
Dat_3(2)-4	**6**
Dat_4(2)-4	0
Dat_5(2)-4	2
Dat_6(2)-4	**7**
Dat_7(2)-4	**5**
Dat_8(2)-4	**5**
Dat_9(2)-4	**7**

**Table 6:** The similar parameters for the sensors 1–3. The sensor 1 is selected as the reference one.

Parameters	CP_1_	CP_2_	CP_3_	CP_4_	CP_5_	CP_6_
Av_dat_1-1	2.00000	2.00000	1.00000	1.00000	0.00000	0.00000
Av_dat_1-3	1.99003	1.99831	0.99755	0.99990	0.99991	0.02523
Dat_0(3)-1	1.74616	1.95858	0.85306	0.90528	0.00000	0.55612
Dat_1(3)-1	1.76948	1.98327	0.82897	0.85659	0.99358	0.52659
Dat_2(3)-1	1.78993	1.98334	0.83776	0.87725	0.99896	0.52485
Dat_3(3)-1	**1.99544**	**1.99565**	**0.96327**	**0.98647**	0.95587	**0.30900**
Dat_4(3)-1	1.74988	1.96089	0.86640	0.91007	0.00799	0.55524
Dat_5(3)-1	1.89731	**1.99238**	**0.98029**	**0.97867**	0.99978	**0.33086**
Dat_6(3)-1	**1.95304**	**1.99429**	**0.97197**	**0.98523**	0.99753	**0.31062**
Dat_7(3)-1	1.77347	1.98065	0.77719	0.85356	1.00000	0.55825
Dat_8(3)-1	**1.92388**	1.99132	0.90513	0.96582	0.97290	0.38061
Dat_9(3)-1	**1.96922**	**1.99437**	**0.94730**	**0.98235**	0.99679	**0.32916**

**Table 7:** Additional correlation parameters for sensors 1–3 that were extracted from complex moments.

Parameters	CP_7_	CP_8_	CP_9_	CP_10_	CP_11_	CP_12_
Av_dat_1-1	2.00000	2.00000	2.00000	2.00000	0.00000	0.00000
Av_dat_1-3	1.98055	1.98136	1.99998	1.92022	0.03030	1.23588
Dat_0(3)-1	1.69569	1.96787	1.88438	**1.99307**	**0.22103**	**0.67753**
Dat_1(3)-1	1.48682	1.98256	1.79888	1.94699	0.35405	1.06661
Dat_2(3)-1	1.49624	**1.98947**	1.80194	1.92174	0.33250	1.01893
Dat_3(3)-1	**1.92799**	1.97649	**1.99932**	1.96534	**0.21478**	0.81327
Dat_4(3)-1	1.69850	1.96966	1.89341	1.89286	**0.19699**	**0.74544**
Dat_5(3)-1	**1.89501**	1.96770	**1.99855**	1.95234	0.23908	**0.75165**
Dat_6(3)-1	**1.99082**	**1.99202**	**1.99440**	1.94151	**0.23857**	0.85859
Dat_7(3)-1	1.44803	**1.98635**	1.77841	**1.98673**	0.34385	0.83632
Dat_8(3)-1	1.75915	1.97309	1.90003	**1.98770**	0.25560	0.97468
Dat_9(3)-1	**1.86734**	**1.99604**	**1.98785**	**1.97617**	0.24269	**0.79408**

**Table 8:** The selection of the “best” sensor among sensors numbered 3. The sensor 1 is kept as the reference.

The Available Set of the Selected Pressure Sensors between 3-1	Number of Optimal Parameters
Dat_0(3)-1	3
Dat_1(3)-1	0
Dat_2(3)-1	1
Dat_3(3)-1	**8**
Dat_4(3)-1	2
Dat_5(3)-1	**7**
Dat_6(3)-1	**9**
Dat_7(3)-1	2
Dat_8(3)-1	2
Dat_9(3)-1	**10**

## Data Availability

The sensor’s measured data can be sent to a potential reader under his personal request.

## Abbreviations

ECU	Electronic control unit
GMV-function	Generalized mean value function
GPCF	Generalized Pearson correlation function
LTFCT	Long-term fuel correction time
OBD-II	On board diagnostics, 2nd generation
PCC	Pearson correlation coefficient
STFCT	Short-term fuel correction time
